# Outcomes and prognosis of progressive pulmonary fibrosis in patients with antineutrophil cytoplasmic antibody-positive interstitial lung disease

**DOI:** 10.1038/s41598-023-45027-0

**Published:** 2023-10-17

**Authors:** Susumu Sakamoto, Aika Suzuki, Sakae Homma, Yusuke Usui, Hiroshige Shimizu, Muneyuki Sekiya, Shion Miyoshi, Yasuhiko Nakamura, Naohisa Urabe, Takuma Isshiki, Atsuko Kurosaki, Kazuma Kishi

**Affiliations:** 1 Division of Respiratory Medicine, Toho University Omori Medical Center, 6-11-1, Ota-Ku Omori Nisi, Tokyo, 143-8541 Japan; 2 Department of Diagnostic Radiology, Fukujuji Hospital, Kiyose, Tokyo, Japan

**Keywords:** Respiratory tract diseases, Autoimmune diseases

## Abstract

Approximately one-third of fibrosing interstitial lung diseases exhibit progressive pulmonary fibrosis (PPF), a clinicopathological condition distinct yet resembling idiopathic pulmonary fibrosis (IPF). PPF in ANCA-positive ILD (ANCA-ILD) is poorly documented. To clarify incidence, predictors of PPF in ANCA-ILD, and their prognostic impact, 56 patients with ANCA-ILD were followed for ≥ 1 year (April 2004 to April 2021). PPF was defined per ATS/ERS/JRS/ALAT PPF 2022 guideline. We compared PPF and non-PPF in 38 patients with pulmonary function tests and ≥ 1 year follow up. ANCA-ILD (19 male, 19 female; mean age 72 years) comprised 21 patients with microscopic polyangiitis ILD (MPA-ILD) and 17 with ANCA-positive IP without systemic vasculitis (ANCA-IP). PPF occurred in 15/38 (39.5%) overall, and 27% of patients with MPA-ILD and 53% with ANCA-IP. Patient characteristics did not differ between PPF and non-PPF, however, the survival was significantly worse in patients with PPF than those with non-PPF. On multivariate regression analysis, higher age, higher serum SP-D level, and lower baseline %FVC were associated with PPF. In ANCA-ILD, 39.5% of patients demonstrated PPF, which is associated with increased mortality. Predictors of PPF were older age, higher SP-D, and lower baseline %FVC.

## Introduction

Interstitial lung disease (ILD) associated with connective tissue disease (CTD) is a heterogeneous condition, which adversely affects quality of life and is associated with increased mortality^1,2^. Antineutrophil cytoplasmic antibody (ANCA)-associated vasculitis (AAV), a group of necrotizing vasculitis predominantly affecting small vessels, can be classified as antibody-specific subtypes into myeloperoxidase-specific ANCA (MPO-ANCA) and proteinase 3 ANCA (PR3-ANCA)^3^. Pulmonary involvement is seen in a substantial percentage of patients typically manifesting as abnormal findings on high-resolution computed tomography (HRCT). In patients with the clinical subtype microscopic polyangiitis (MPA), diffuse alveolar hemorrhage and ILD are a well-known form of pulmonary involvement^4^. Moreover, among East Asian populations, including the Japanese, MPA and MPO-ANCA positive AAVs are more prevalent than in Europeans, and ILD is more frequent than pulmonary hemorrhage in MPA^5–10^. Conversely, some patients have ANCA-positive interstitial pneumonia (IP) but no apparent systemic vasculitis (ANCA-IP); 10–30% of these patients develop systemic vasculitis during the clinical course^11,12^.

A subset of patients develops progressive pulmonary fibrosis (PPF), which is characterized by deteriorating symptoms, declining pulmonary function, and/or progressive fibrosis on high-resolution computed tomography (HRCT)^13–16^. Progressive fibrosing interstitial lung diseases (PF-ILDs) have been defined in INBUILD^®^, a randomized, double-blind, placebo-controlled trial on chronic fibrosing ILDs with a progressive course^17^. They have been reported variously in ILDs such as collagen vascular disease, hypersensitivity pneumonitis (HP), and idiopathic interstitial pneumonias (IIPs) of the fibrosing type other than IPF (non-IPF). About one-third of fibrosing ILDs (fILDs) have a clinicopathological behavior resembling Idiopathic pulmonary fibrosis (IPF) demonstrating a progressive course. PF-ILD is associated with decreased quality of life and increased mortality risk^17^.

The updated 2022 American Thoracic Society/European Respiratory Society/Japanese Respiratory Society/Asociación Latinoamericana de Tórax (ATS/ERS/JRS/ALAT) IPF guidelines defined progressive pulmonary fibrosis (PPF) as a feature of ILD in non-IPF disease with progressive deterioration^18^.

However, there is a paucity of reports on PPF in ANCA-positive ILD (ANCA-ILD), including MPA-ILD and ANCA-IP. Clinical characteristics of patients with PPF in ANCA-ILD remain unknown. Thus, this study aimed to assess the incidence and predictors of PPF in ANCA-ILD and to evaluate the impact of clinical phenotype on survival.

## Methods

### Study subjects

**Figure 1:**
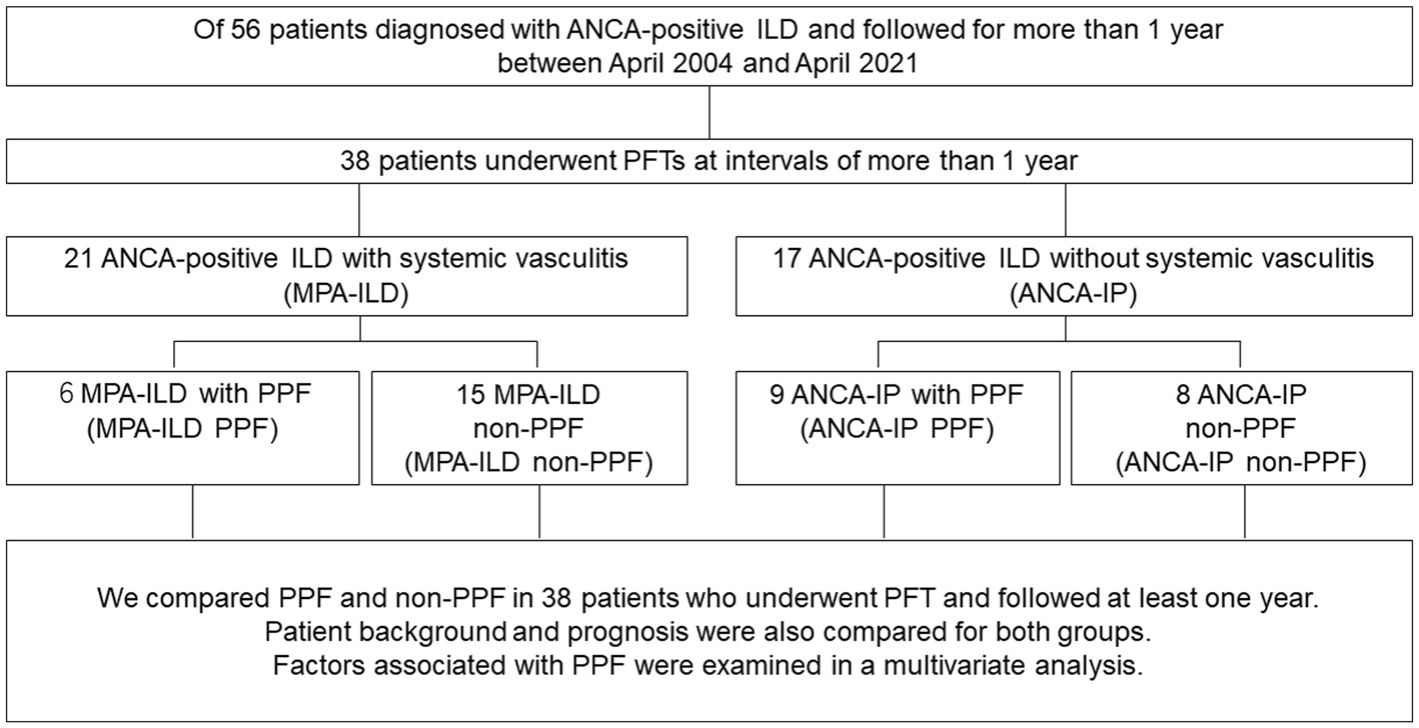
Study flowchart.

This single-center retrospective cohort study at Toho University Omori Medical Center included 56 eligible patients diagnosed with ANCA-ILD with a minimum follow-up of 1 year between April 2004 and April 2021. Overall, 38 patients underwent PFTs at intervals of at least 1 year. ANCA-ILD was defined as serum ANCA positivity with ILD. Among the patients with ANCA positive-ILD, those with systemic vasculitis were classified as MPA-ILD and those with ANCA positivity alone and ILD but with no systemic vasculitis were classified as ANCA-positive IP (ANCA-IP) (Fig. 1).

An MPA diagnosis was made by using an algorithm for primary systemic vasculitis as proposed by the European Medicines Agency^19^.

### Data collection

Demographic, clinical, radiological, and PFT data including % predicted forced vital capacity (%FVC), and lung diffusing capacity for carbon monoxide (DL_CO_) % predicted were retrieved from medical records. Data also included age, sex, obesity, disease duration, and smoking history (pack-years). Past and current treatment history were also documented.

### Laboratory results

Results of the following laboratory investigations were collected from medical records: C-reactive protein (CRP), Krebs von den Lungen-6 (KL-6), surfactant protein D (SP-D), PaO_2_, and MPO-ANCA titer.

### Chest imaging

For all patients, HRCT images were acquired at diagnosis, regardless of respiratory symptoms. HRCT was performed pretreatment with follow up at 6 and/or 12 months thereafter.

All HRCT images were reviewed by 4 pulmonologists (S.H., K.S., A.S., and Y.U.) and 1 specialist pulmonary radiologist (A.K.) who were blinded to patient clinical and laboratory findings. Judgements were compared relative to clinical features and laboratory findings for each patient. All scans were acquired during inspiration with the patient supine; slice thickness was ≤ 1.0 mm at 10–20-mm intervals from the apices to the lung bases. IP was classified as usual interstitial pneumonia (UIP), probable UIP, indeterminate for UIP, and alternative diagnosis according to the 2022 updated ATS/ERS/JRS/ALAT PPF guidelines^18^.

We evaluated serial changes in HRCT findings before and at 6, 12, and 24 months. Using the PPF guidelines, disease progression was designated as the following findings on HRCT within the past year: (1) Increased extent or severity of traction bronchiectasis and bronchiectasis; (2) New ground-glass opacity with traction bronchiectasis: (3) New fine reticulation; (4) Increased extent or increased coarseness of reticular abnormality; (5) New or increased honeycombing; and (6) Increased lobar volume loss.

### Pulmonary function tests

Lung volumes, forced expiratory volume in 1 s, and DL_CO_ were measured by using the Chestac-8800 automated pulmonary testing system (Chest Co. Ltd., Tokyo, Japan) according to standard methods. Values were expressed as percentages of the predicted value. Data at baseline, 6 months, 1 year, 2 years, and last follow up were recorded. Serial PFT trends at 1 year, expressed as percentages of baseline values, were evaluated for FVC.

### Definition of progressive pulmonary fibrosis (PPF)

Briefly, PPF was defined based on the 2022 ATS/ERS/JRS/ALAT PPF guideline^18^.

In a patient with ILD of known or unknown etiology other than IPF and overt radiological evidence of pulmonary fibrosis, PPF is defined as at least two of the following three criteria occurring within the past 1 year with no alternative explanation: (1) Worsening respiratory symptoms; (2) Physiological evidence of disease progression defined as: (i) absolute decline in FVC ≥ 5% predicted within 1 year of follow-up or (ii) absolute decline in DL_CO_ (corrected for Hb) ≥ 10% predicted within 1 year of follow-up; and (3) Radiological evidence of disease progression defined as: (a) increased extent or severity of traction bronchiectasis and bronchiolectasis; (b) new ground-glass opacity with traction bronchiectasis; (c) new fine reticulation; (d) increased extent or increased coarseness of reticular abnormality; (e) new or increased honeycombing; and (f) increased lobar volume loss^18^.

We compared PPF and non-PPF in 38 patients who underwent PFTs and were followed at least 1 year with comparison for patient background and prognosis/outcome for both groups. Additionally, multivariate analysis was performed to examine factors associated with PPF.

Finally, we performed a survival analysis to compare idiopathic interstitial pneumonias (IIPs) and ANCA-ILD, with similar HRCT patterns. Using data from the IIPs cohort at our institution, 63 patients with IPF and 79 with idiopathic nonspecific interstitial pneumonia (iNSIP) were identified and compared to those with ANCA-ILDs (ANCA-UIP, ANCA-NSIP). We also evaluated the incidence of PPF and compared this among these groups.

### Statistical analysis

Continuous variables are expressed as median (range), unless otherwise stated; comparison was made by using the Mann–Whitney U test. Categorical variables were compared by using the χ2 test. A P value of < 0.05 was considered to indicate statistical significance. All statistical analyses were performed by using SPSS version 11.0 (SPSS Inc., Chicago, IL).

### Ethical approval

This retrospective study was conducted in accordance with the amended Declaration of Helsinki and was approved by the Ethics Committee of Toho University Omori Medical Center in April 2022 (project approval number M20221). Considering the retrospective design, the requirement for informed consent was waived by Ethics Committee of Toho University Omori Medical Center because of anonymized patient data. The study protocol was in accordance with relevant guidelines.

## Results

### Patient characteristics

In total, 56 patients with ANCA-ILD were identified and included. Of these, 38 patients (19 men and 19 women; mean age 72 years) underwent routine PFTs at intervals of over 1 year. Their characteristics are shown in Table 1. This patient cohort comprised 21 with MPA-ILD and 17 with interstitial pneumonia with ANCA positivity alone with no systemic vasculitis (ANCA-IP). The patients were then divided into MPA-ILD and ANCA-IP groups. Age, sex, and smoking history did not differ between MPA-ILD and ANCA-IP. Evaluation of both UIP and probable UIP patterns as UIP patterns showed a significantly higher proportion of UIP patterns for ANCA-IP than for MPA-ILD (65% vs 29%, P = 0.03). All MPA-ILD patients (n = 21) were treated with high-dose (> 0.6 mg/kg) glucocorticoids (GC). Of these, 16 received immunosuppressant therapy; only 1 received antifibrotic agents. In contrast, in the ANCA-IP group (n = 17), 10 patients were treated with low dose (≤ 0.5 mg/kg) GC, 2 were treated with antifibrotic agents, while 6 patients did not receive any treatment.

**Table 1:** Baseline clinical characteristics and treatment in patients with MPA-ILD and ANCA-IP.

	MPA-ILD (n = 21)	ANCA-IP (n = 17)	Total (n = 38)	P value
Age	71.3 ± 8.5	72.1 ± 11.3	71.7 ± 7.8	0.908
Male sex n (%)	10 (48%)	9 (53%)	19 (50%)	0.774
Smoking n (%)	11 (52%)	11 (81%)	22 (58%)	0.444
Smoking index	329.5 ± 106.1	466.8 ± 28.3	390.9 ± 594.0	0.383
HRCT pattern	(UIP/probable/indeterminate/alternative diagnosis)			
	3/3/6/9	7/4/3/3	10/7/9/12	
UIP pattern n (%)	6 (29%)	11 (65%)	17 (45%)	0.026*
CRP [mg/dL]	5.1 ± 13.6	1.8 ± 0.4	3.4 ± 13.4	0.352
KL-6 [U/mL]	948.5 ± 2021.7	992.4 ± 722.2	968.1 ± 1743.0	0.399
SP-D [ng/mL]	190 ± 198.1	172.7 ± 360.3	182.3 ± 373.4	1.000
MPO-ANCA [U/mL]	375.3 ± 66.5	71.7 ± 11.5	261.4 ± 4.5	0.006*
PaO_2_ [Torr]	86.7 ± 4.7	81.6 ± 11.4	84.3 ± 17.3	0.379
FVC [L]	2.37 ± 0.53	2.55 ± 1.43	2.45 ± 1.86	0.179
%FVC [%]	88.6 ± 24.1	92.5 ± 28.0	90.3 ± 33.4	0.383
%DL_CO_ [%]	66.7 ± 8.1	69.8 ± 15.5	68.3 ± 15.1	0.709
PPF n (%)	6 (29%)	9 (53%)	15 (39.5%)	0.126
Treatment				
High-dose GC n (%)	21(100)	0 (0)	21 (55.3)	< 0.001
Low-dose GC n (%)	0 (0)	19 (58.8)	19 (50.0)	< 0.001
Immunosuppressant n (%)	16 (76.2)	1 (5.9)	17 (44.7)	0.002*
Antifibrotic agent n (%)	1 (4.8)	2 (11.8)	3 (7.9)	0.577
None	0 (0)	6 (35.3)	6 (15.8)	0.003*

*MPA* microscopic polyangiitis, *ANCA* antineutrophil cytoplasmic antibody, *IP* interstitial pneumonia, *HRCT* high-resolution computed tomography, *UIP* usual interstitial pneumonia, *CRP* C-reactive protein, *KL-6* Krebs von den Lungen-6, *SP-D* surfactant protein D, *FVC* forced vital capacity, *DL*_*CO*_ carbon monoxide diffusing capacity, *PPF* progressive pulmonary fibrosis, *GC* glucocorticoid.

*P < 0.05.

### Laboratory investigations and pulmonary function test (PFT) results

Laboratory results for MPA-ILD and ANCA-IP are also shown in Table 1. Serum MPO-ANCA levels were significantly higher in the MPA-ILD compared to ANCA-IP group (375.3 vs. 71.7 U/mL, P = 0.006). Serum CRP level tended to be higher in MPA-ILD than ANCA-IP, but was not significant (5.1 vs. 1.8 mg/dL, P = 0.352). Levels of serum ILD biomarkers such as KL-6 and SP-D, did not differ between the two groups (948.5 vs. 992.4 U/mL, P = 0.399; 190.0 vs. 172.7 ng/mL, P = 1.000). For PFTs, mean baseline FVC and %FVC did not differ between the two groups (2370 vs. 2550 mL P = 0.179, 88.6 vs. 92.5%, P = 0.383). Mean baseline %DL_CO_ did not differ between the two groups (66.7 vs. 69.8%, P = 0.709). All MPA-ILD patients received high-dose GC and/or immunosuppressants like cyclophosphamide. Also, various treatments were administered in the ANCA-IP group depending on the HRCT pattern and/or pathologic pattern including low-dose GC, immunosuppressants, and/or antifibrotic agents. Use of high-dose GC and immunosuppressant therapy was significantly higher in the MPA-ILD vs ANCA-IP group (100 vs 0%, P < 0.001; 76.2 vs. 5.9%, P = 0.002). Use of antifibrotic agents did not differ between both groups (4.8 vs. 11.8%, P = 0.577). The proportion of patients who received no treatment was significantly lower in MPA-ILD than in ANCA-IP (0 vs. 35.3%, P = 0.003).

### Incidence of PPF

**Figure 2:**
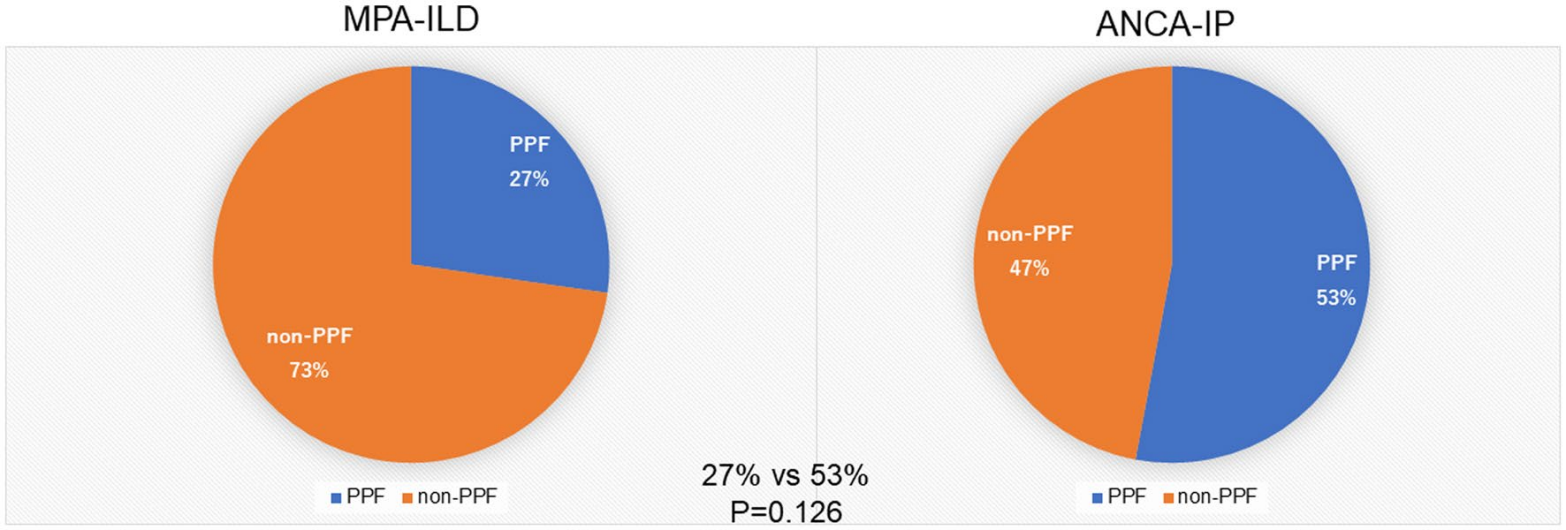
Incidence of PPF in MPA-ILD and ANCA-IP cohorts.

**Figure 3:**
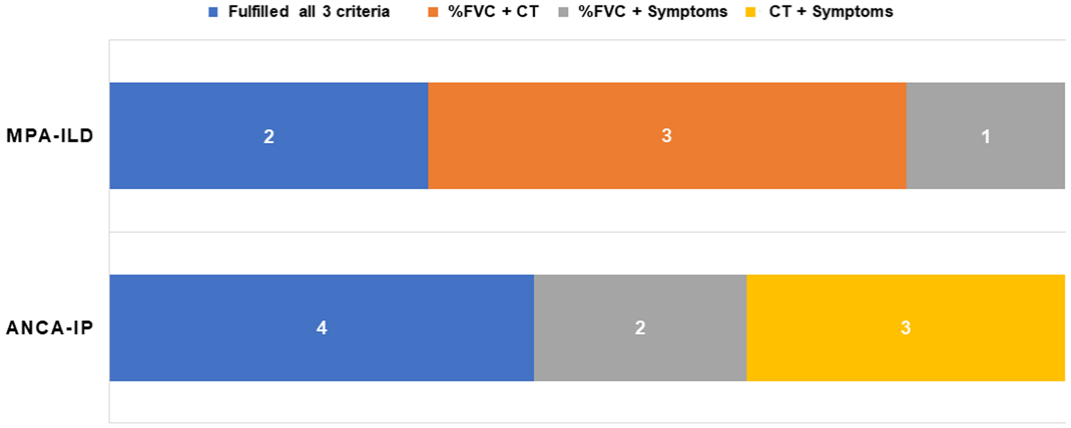
Clinical features of PPF.

In the ANCA-ILD cohort (n = 38) 15 (39.5%) patients developed PPF during follow up according to the international criteria defined in the “Methods” section. Of these, 6 of 21 (27%) MPA-ILD and 9 of 17 (53%) ANCA-IP demonstrated PPF. The incidence of PPF tended to be higher in ANCA-IP than MPA-ILD but did not differ significantly (53% vs 27%, P = 0.126) (Fig. 2). Clinical features of PPF were: decreasing %FVC ≥ 5% in 1 year, deterioration of HRCT findings, and worsening subjective symptoms in 6 patients (40.0%) who fulfilled all 3 criteria; %FVC decline ≥ 5% in 1 year and deterioration of HRCT findings were seen in 3 patients (20%). %FVC decline ≥ 5% per year and worsening subjective symptoms were seen in 3 patients (20%); and deteriorating HRCT findings and subjective symptoms in 3 patients (20%). In the MPA-ILD cohort, 2 patients fulfilled all 3 criteria, 3 fulfilled %FVC and HRCT, and 1 fulfilled %FVC and subjective symptoms criteria. For the ANCA-IP cohort, 4 patients fulfilled all 3 criteria, 2 fulfilled %FVC and subjective symptoms, and 3 fulfilled HRCT and subjective symptoms criteria (Fig. 3).

### Comparison of clinical features between PPF and non-PPF

Table 2 shows patient characteristics, laboratory findings, and PFT results for patients with PPF and non-PPF disease. Age, sex, and smoking history did not differ between the groups. Serum MPO-ANCA and CRP levels did not differ between the groups (55.2 vs. 327.7 U/mL, P = 0.26; 2.3 mg/dL vs. 4.2 mg/dL P = 0.36). Serum KL-6 and SP-D levels tended to be higher in PPF than non-PPF; the difference was not significant (1158 vs. 843 U/mL, P = 0.425; 253.1 vs. 120.2 ng/mL, P = 0.068). Regarding PFTs, FVC and %FVC tended to be lower in the PPF patients than non-PPF but the differences were not significant (2370 mL vs. 2550 mL, P = 0.145; 88.6% vs. 92.5%, P = 0.121). Also, %DL_CO_ did not differ between the two groups (66.7% vs. 69.8%, P = 0.845). Together, no significant baseline clinical differences were demonstrated when comparing PPF and non-PPF patients.

**Table 2:** Baseline clinical characteristics and treatments in PPF and non-PPF groups.

	PPF (n = 15)	Non-PPF (n = 23)	Total (n = 38)	
Age	74.5 ± 8.4	69.8 ± 8.0	71.7 ± 7.8	0.089
Male sex n (%)	11 (73%)	8 (35%)	19 (50%)	0.740
Smoking n (%)	11 (73%)	11 (48%)	22 (58%)	0.258
Smoking index	409.0 ± 514.7	379.1 ± 330.0	390.9 ± 594.0	0.976
MPA-ILD n (%)	6 (40%)	15 (65%)	21 (55%)	0.126
UIP pattern n (%)	7 (47%)	10 (43%)	17 (45%)	0.847
CRP [mg/dL]	2.3 ± 5.6	4.2 ± 6.2	3.4 ± 13.4	0.359
KL-6 [U/mL]	1158.7 ± 1002.3	843.9 ± 704.6	968.1 ± 1743.0	0.425
SP-D [ng/mL]	253.1 ± 227.6	120.2 ± 89.3	182.3 ± 373.4	0.068
MPO-ANCA [U/mL]	55.2 ± 82.7	327.7 ± 683.7	261.4 ± 4.5	0.260
PaO_2_ [Torr]	84.0 ± 16.0	84.5 ± 10.3	84.3 ± 17.3	0.665
FVC [L]	2.25 ± 0.83	2.58 ± 0.67	2.45 ± 1.86	0.145
%FVC [%]	83.1 ± 22.6	95.0 ± 16.8	90.3 ± 33.4	0.121
%DL_CO_ [%]	68.5 ± 16.0	68.1 ± 17.2	68.3 ± 15.1	0.845

*MPA* microscopic polyangiitis, *ANCA* antineutrophil cytoplasmic antibody, *IP* interstitial pneumonia, *HRCT* high-resolution computed tomography, *UIP* usual interstitial pneumonia, *CRP* C-reactive protein, *KL-6* Krebs von den Lungen-6, *SP-D* surfactant protein D, *FVC* forced vital capacity, *DL*_*CO*_ carbon monoxide diffusing capacity, *PPF* progressive pulmonary fibrosis, *GC* glucocorticoid.

*P < 0.05.

### Predictors of PPF in patients with ANCA-ILD

Correlations between clinical factors, pulmonary function, HRCT findings, and occurrence of PPF were examined. Univariate regression analysis showed higher serum level of SP-D was associated with PPF (OR 1.006 95% CI 1.000–1.012) (Table 3). On multivariate regression analysis, higher age, higher serum SP-D level, and lower %FVC were associated with PPF (OR 1.223 95% CI 1.036–1.468, OR 1.012 95% CI 1.003–1.022, OR 0.949 95% CI 0.905–0.996).

**Table 3:** Correlations of clinical factors, pulmonary function, and HRCT findings, with PPF.

	Odds ratio	95% CI	P value
Univariate regression analysis			
Age	1.079	0.987–1.180	0.093
Male sex	1.247	0.339–4.589	0.740
MPA	0.356	0.093–1.362	0.131
Smoking	0.467	0.124–1.762	0.261
Smoking index	1.000	0.999–1.002	0.823
PaO_2_	0.997	0.944–1.052	0.911
KL-6	1.000	1.000–1.001	0.269
SP-D	1.006	1.000–1.012	0.040*
CRP	0.942	0.830–1.069	0.353
MPO-ANCA	0.996	0.990–1.001	0.131
UIP pattern on HRCT	1.137	0.308–4.204	0.847
Corticosteroid use	1.187	0.225–6.260	0.839
FVC	0.508	0.186–1.385	0.185
%FVC	0.967	0.932–1.004	0.081
%DL_CO_	1.001	0.960–1.045	0.946
Multivariate regression analysis			
Age	1.233	1.036–1.468	0.019*
SP-D	1.012	1.003–1.022	0.010*
%FVC	0.949	0.905–0.996	0.032*

*MPA* microscopic polyangiitis, *ANCA* antineutrophil cytoplasmic antibody, *IP* interstitial pneumonia, *HRCT* high-resolution computed tomography, *UIP* usual interstitial pneumonia, *CRP* C-reactive protein, *KL-6* Krebs von den Lungen-6, *SP-D* surfactant protein D, *FVC* forced vital capacity, *DL*_*CO*_ lung carbon monoxide diffusing capacity, *PPF* progressive pulmonary fibrosis, *GC* glucocorticoid.

*P < 0.05.

### Survival analysis

**Figure 4:**
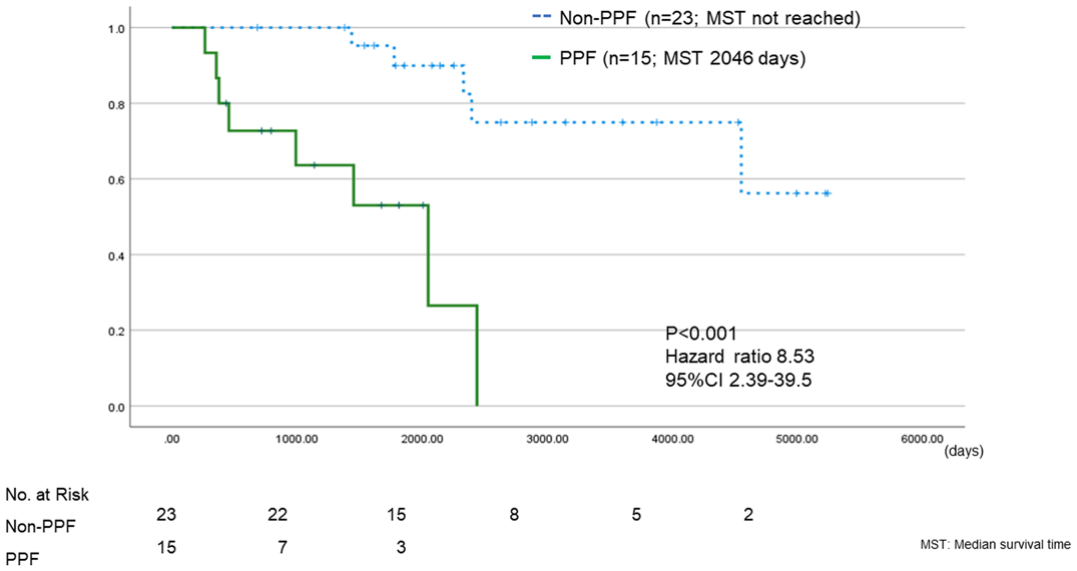
Kaplan–Meier plots for survival analysis from time of diagnosis among PPF and non-PPF groups.

**Figure 5:**
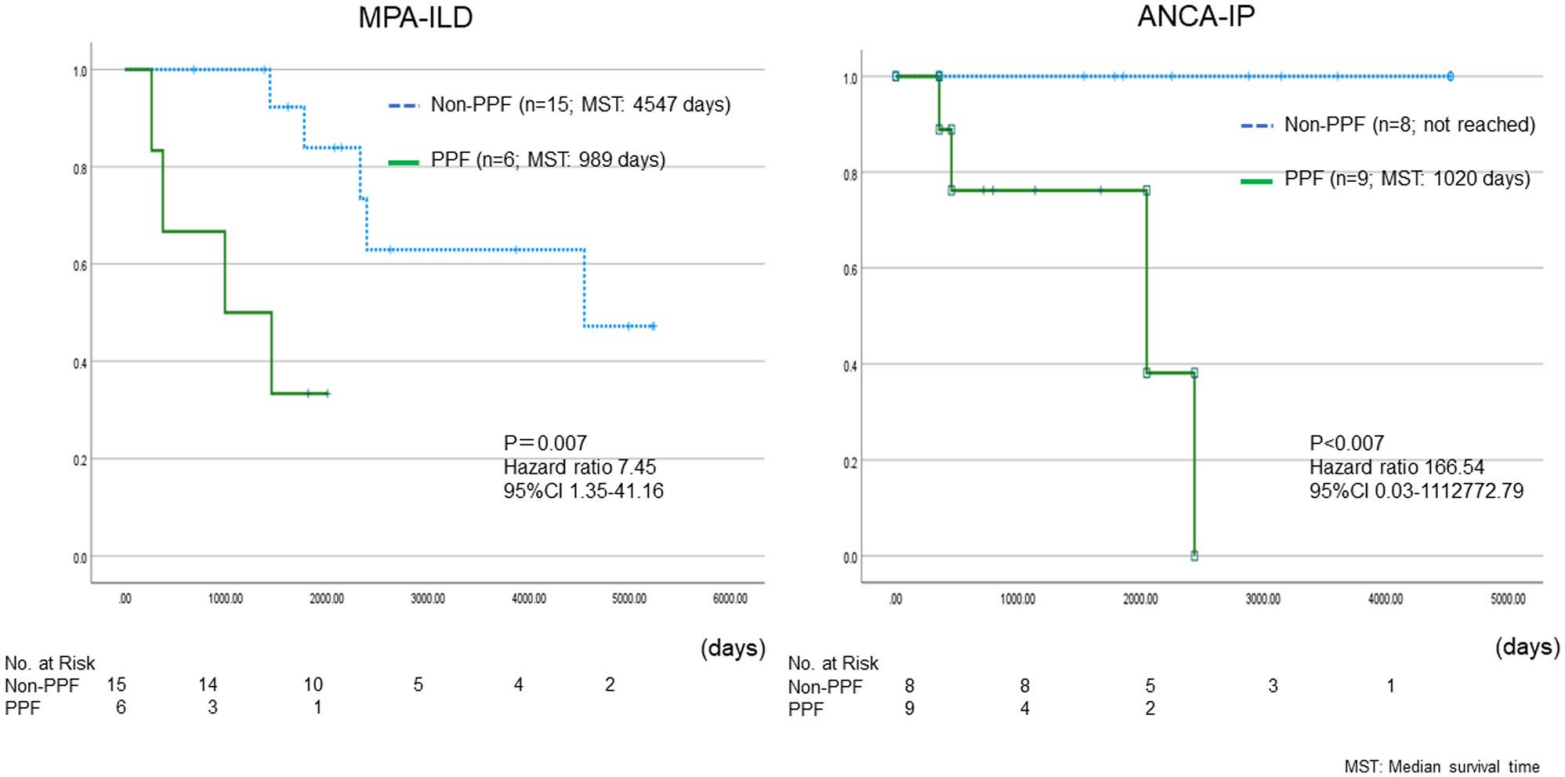
Kaplan–Meier plots for survival analysis from time of diagnosis divided into MPA-ILD and ANCA-IP among PPF and non-PPF groups.

The Kaplan–Meier curve for survival from the time of diagnosis is shown in Fig. 4. Prognosis was significantly worse for PPF than non-PPF in the overall population [Median survival time (MST) 2046 days vs not reached, Hazard ratio (HR) 8.53, 95% confidence interval (CI) 2.39–29.5, P < 0.001]. When ANCA-ILD was categorized into MPA-ILD and ANCA-IP, prognosis was also worse for PPF in both groups (MPA-ILD: MST 989 vs 4547 days, HR 7.45, 95% CI 1.35–41.16, P = 0.007; ANCA-IP: MST 1020 days vs not reached, HR 166.54, 95% CI 0.03–1,112,772.79, P = 0.007) (Fig. 5).

### Survival and incidence of PPF compared between idiopathic interstitial pneumonias and ANCA-ILD with similar HRCT pattern

**Figure 6:**
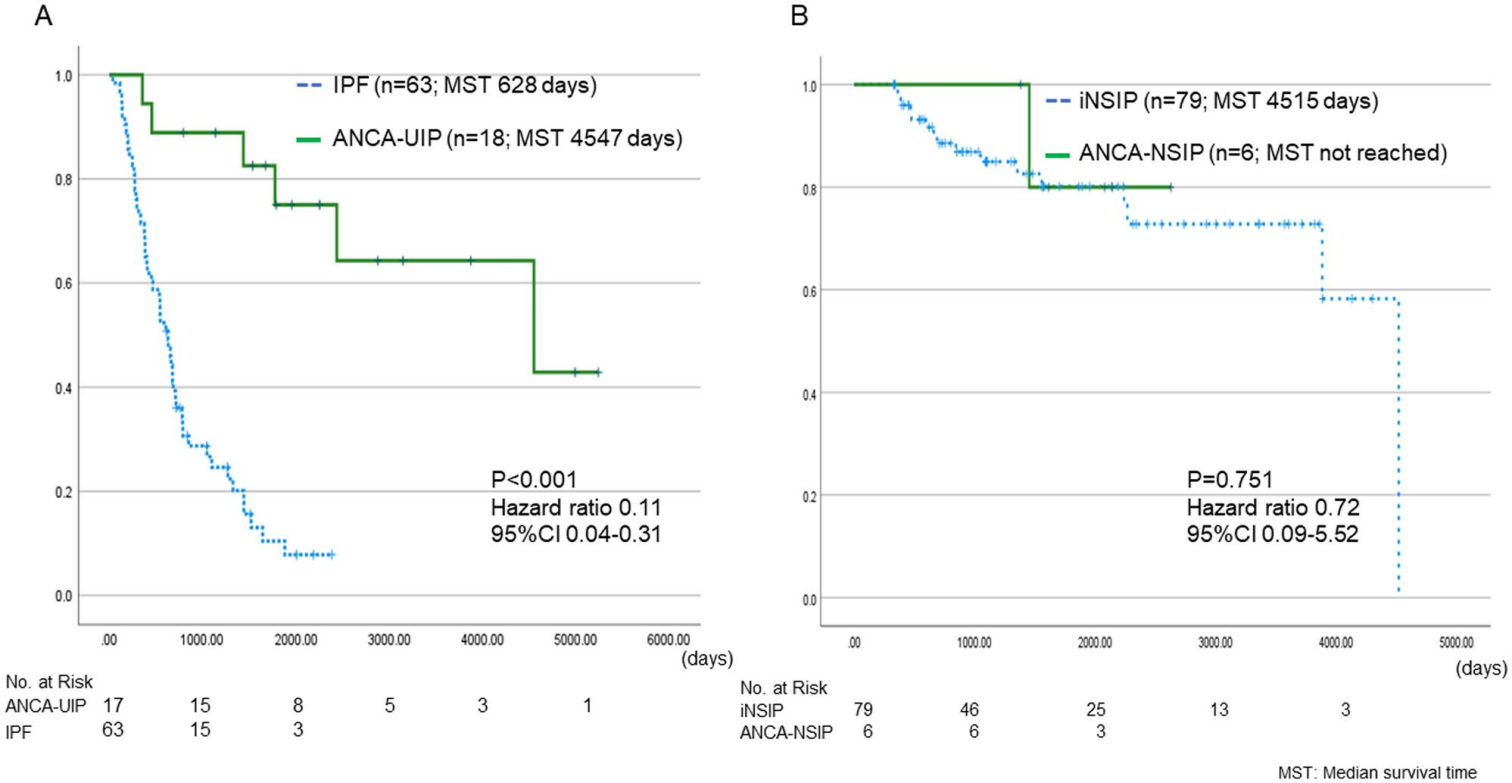
Kaplan–Meier plots for survival analysis from time of diagnosis in idiopathic interstitial pneumonias and ANCA-ILD with similar HRCT pattern. (**A**) ANCA-UIP vs IPF. (**B**) ANCA-NSIP vs idiopathic NSIP.

Survival rate was compared between idiopathic interstitial pneumonias (IIPs) and ANCA-ILD with similar HRCT pattern. Prognosis was significantly better for ANCA-ILD with usual interstitial pneumonia (UIP) pattern on HRCT (ANCA-UIP) than IPF (MST 4547 vs 628 days, HR 0.11, 95% CI 0.04–0.31, P < 0.001) (Fig. 6A). The incidence of patients developing PPF was significantly lower in ANCA-UIP than for IPF (41.1% vs 66.7% P = 0.04). Otherwise, no significant differences in terms of survival were found in the ANCA-NSIP and iNSIP (MST 4515 days vs not reached, HR 0.72, 95% CI 0.09–5.52, P < 0.751) (Fig. 6B). The incidence of patients developing PPF did not differ between ANCA-NSIP and iNSIP (16% vs. 21.5% P = 0.79).

## Discussion

To our knowledge, a few previous studies have investigated serial changes in clinical symptomatology, PFT results, and HRCT findings in patients with MPA-ILD or ANCA-IP^20,21^. Our study found a relatively high incidence where approximately 40% of patients with ANCA-ILD demonstrated PPF. Among the ANCA-ILD cohort, PPF was more frequent in ANCA-IP than in MPA-ILD.

Cases with FVC decline over time and worse prognosis in IPF have been reported^1^. Around one-third of fILDs have a clinical disease behavior similar to IPF, demonstrating a PPF in those patients with IIPs, CTD-ILD, HP, and conditions other than IPF, and these patients have a worse prognosis. The concept of PF-ILD was proposed in the INBUILD trial^17^ against this background and was defined as PPF in the updated 2022 IPF guideline^18^. The clinical entity of PPF describes a high-risk population in patients with fILDs and manifests as deterioration in pulmonary symptoms, HRCT findings, and pulmonary function.

We identified that older age, higher serum SP-D level, and lower %FVC at baseline were clinical predictors of PPF in ANCA-ILD. It has been shown that older age^22^, higher serum SP-D level^23^, and lower %FVC^24^ at baseline were associated with worse outcome in patients with IPF. In another report, fibrosis HRCT pattern at baseline, diabetes mellitus, and steroid-use posed a higher risk of development of PF-ILD in patients with CTD-ILD^25^. Furthermore, our study demonstrated an association of PPF with increased mortality in patients with ANCA-ILD. Our results are consistent with the previous INBUILD trial which identified patients with PF-ILD as a high-risk group for worse outcome^17^. In the INBUILD trial, UIP-like pattern on HRCT among patients with non-IPF fibrotic ILD was associated with greater FVC decline^17^. In this study, however, the proportion of UIP patterns on HRCT was significantly higher for ANCA-IP than for MPA-ILD; UIP pattern was not associated with PPF. These findings might be due to insufficient sample size, and we need to accumulate more cases to confirm these findings. Takakuwa et al. investigated survival and prognostic factors in patients with ANCA-ILD and found survival was significantly lower in patients with UIP pattern than those with the NSIP pattern^20^. According to Libra et al., evaluating the role of p-ANCA in predicting clinical evolution and prognosis in a cohort of IPF patients revealed similar lung function decline in IPF patients with and without p-ANCA during follow-up. However, IPF p-ANCA + showed better survival^21^. Similarly, our study showed that the survival was better in ANCA-UIP than IPF. These results indicate potential behavioral similarity of ANCA-UIP and UIP associated with collagen vascular disease.

Although, the efficacy of nintedanib in PF-ILD for patients with non-IPF disease was reported in the INBUILD trial, there is no similar evidence for ANCA-ILD. To date, the role of anti-inflammatory or anti-fibrotic agents in ANCA-ILD has not been evaluated. In our study, all MPA-ILD patients received high-dose corticosteroids and immunosuppressants such as cyclophosphamide. However, 27% of MPA-ILD patients developed PPF despite immunosuppressant therapy. Therefore, the efficacy of antifibrotic agents in patients with PPF despite use of anti-inflammatory agents in MPA-ILD needs to be examined. Nevertheless, the ANCA-IP cohort received various treatments determined by the specific HRCT and/or pathologic pattern. These medications included corticosteroids, immunosuppressants, and/or antifibrotic agents in spite of which 53% of ANCA-IP developed PPF.

To date, there is no established treatment protocol for patients with ANCA-IP. Several studies have demonstrated the role of immunosuppressants in reducing the risk of developing MPA in MPO-ANCA-positive patients initially diagnosed with IPF^11,12,25^. Specifically, patients who do not receive immunosuppressants might be at a relatively high risk of developing MPA. According to Hozumi et al*.*, the absence of immunosuppressant or anti-fibrotic treatment for ANCA-IP was correlated with MPA^26^.

Hosoda et al.^27^ reported significantly increased attenuation areas around honeycombing on chest HRCT in patients with UIP and MPO-ANCA positivity but without MPA (ANCA/UIP). Pathologically, ANCA/UIP is characterized by more prominent inflammatory cell infiltration, lymphoid follicles with germinal centers, and cellular bronchiolitis. Takemura et al. reported that in ANCA-IP with UIP HRCT pattern, patients showed pathological findings of cellular inflammation in interstitial tissue^28^, destructive bronchiolitis, and cysts and that these findings were observed radiologically as honeycombing with increased attenuation around honeycombing. These pathological findings suggest that anti-inflammatory treatment may be effective even in the UIP pattern and should be verified in the future. A prospective study is requisite to validate the clinical effectiveness of immunosuppressant or anti-fibrotic treatment for patients with ANCA-IP and PPF.

Novel developments in endotyping regarding biological understanding of disease and the emerging field of precision medicine have revealed the inadequacy of “a one-size-fits-all approach” in the comprehensive management of chronic lung diseases. Although several reports identified candidate diagnostic or prognostic biomarkers for IPF, impeding factors have hindered the translation of these results into clinical practice^29^. Our findings approximate a step for robust precision medicine approaches in pulmonary fibrosis, potentially highlighting the importance of ANCA endotyping towards an innovative approach in precision medicine. The utility of endotyping with ANCA as a prognostic or theragnostic biomarker in pulmonary fibrosis requires further investigation.

This study has limitations that warrant consideration. First, this was a single-center retrospective study with a small sample size. A large-scale multicenter study is needed to confirm these findings. Second, the study included various HRCT and/or pathologic patterns, which may inadvertently affect the treatment effect and prognosis. Finally, patients with ANCA-IP received various treatments based on HRCT and/or pathologic pattern such as corticosteroids, immunosuppressants, and/or antifibrotic agents. Some patients did not receive any treatment. This variation in treatment regimens may affect the results.

## Conclusion

Among all patients with ANCA-ILD, 39.5% showed features consistent with PPF. PPF is associated with increased mortality in patients with ANCA-ILD. Older age, high SP-D level, and lower %FVC at baseline were associated with PPF in patients with ANCA-ILD. These findings highlight the need for follow-up assessment using PFTs and HRCT in these patients. Studies with a larger patient population and longer follow-up duration are needed to clarify the course of ILD in patients with ANCA-ILD.

## Data Availability

The datasets generated during and/or analyzed during the current study are available from the corresponding author upon reasonable request.
